# *Eucalyptus globulus* and *Cymbopogon flexuosus* essential oils antimicrobial and conservative effects against *Salmonella enterica* serovar typhimurium and possible cytotoxicity: an in vitro and in situ investigation in inoculated pork sirloin

**DOI:** 10.1007/s42770-026-01923-x

**Published:** 2026-04-27

**Authors:** Cleiton Jesus Andrade Pereira, Vithor Parada Garcia, Vitória Rose de Andrade, Milena Mattes Cerveira, Jéssica Silveira Vitoria, David de Andrade Cabral, Eliezer Avila Gandra, Janice Luehring Giongo, Rodrigo de Almeida Vaucher

**Affiliations:** 1 Center of Chemical, Pharmaceutical, and Food Sciences, Research Laboratory in Biochemistry and Molecular Biology of Microorganisms (LAPEBBIOM), Federal University of Pelotas, Pelotas, RS 969010-900 Brazil; 2 Center for Chemical, Pharmaceutical, and Food Sciences, Laboratory of Food Science and Molecular Biology (LACABIM), Federal University of Pelotas, Pelotas, RS 96010-900 Brazil; 3 Faculty of Medicine (FAMED), Pharmacy of Course, Federal University of Rio Grande, Rio Grande, 92203-900 Brazil

**Keywords:** Essential oils, Salmonelosis, Food industry, Conservative effect, Bacterial metabolism

## Abstract

Foodborne illnesses, particularly those caused by *Salmonella* spp., represent a major global health concern, with *S. typhimurium* being a significant contributor to outbreaks. The persistence of *S. typhimurium* in food production environments and its resistance to conventional disinfectants highlight the need for alternative antimicrobial solutions. Essential oils (EOs) from *Eucalyptus globulus* (EGEO) and *Cymbopogon flexuosus* (CFEO) have shown antimicrobial potential against *S. typhimurium*, but their mechanisms of action and cytotoxic effects on eukaryotic cells require further investigation to assess their safety for food applications, particularly in situ. We evaluated cytotoxicity in erythrocytes and zebrafish liver (ZF-L) cells using hemolysis (HA), MTT, and NR uptake assays. Additionally, we investigated the antimicrobial behavior of EGEO and CFEO against *S. typhimurium* ATCC 14,028 through MIC/MBC tests, time-kill assays, and a newly designed TSI-adapted test to assess the EOs’ impact on bacterial metabolism. An in situ experiment was also conducted to evaluate the preservative effects of EGEO and CFEO on pork sirloin contaminated with an infectious dose of *S. typhimurium* and treated with EO-based brines for 7 days under refrigeration. We monitored the presence of *Salmonella* spp., as well as pH, calcium (Ca²⁺), and magnesium (Mg²⁺) concentrations in the brined samples. Results indicated that CFEO was hemolytic above 0.73 mg/mL and EGEO above 0.45 mg/mL. The MTT assay showed that CFEO did not impair mitochondrial function from 2.941 mg/mL and EGEO from 14.36 mg/mL. NR uptake assay showed that both EOs did not impaired lysossomal function in ZF-L. The MICs and MBCs for CFEO and EGEO were 0.37 mg/mL and 1.79 mg/mL, respectively. Time-kill assays revealed bactericidal effects for CFEO at 4x and 2x MIC within 6 h, while EGEO showed a bactericidal effect only at 4x MIC. The EO-TSI agar assay demonstrated that EGEO inhibited sugar fermentation and sulfur metabolism, while CFEO impacted peptone oxidation and protein metabolism. In situ analyses showed CFEO caused more significant changes in color and odor of the pork samples than EGEO, while both maintained pH levels around 6. Ca²⁺ and Mg²⁺ levels were lower in CFEO-treated samples. Microbiological data confirmed the absence of *Salmonella* spp. in all samples except for two in the CFEO-brine group. None of the samples showed signs of autolysis or putrefaction, suggesting that both CFEO and EGEO, particularly EGEO, are feasible alternatives as food preservatives, depending on the desired application.

**Supplementary Information:**

The online version contains supplementary material available at 10.1007/s42770-026-01923-x.

## Introduction

Foodborne illnesses remain a major global public health concern, with bacterial pathogens being one of the leading causes of outbreaks and contamination in food products [1]. Among these, *Salmonella* spp. are particularly significant due to their widespread occurrence, high resilience in diverse environments, and ability to cause severe infections in humans and animals [1–3]. Salmonellosis is a common zoonotic foodborne infection caused by bacteria of the *Salmonella* genus, with millions of cases and substantial mortality worldwide each year [4, 5]. It is the most frequent bacterial foodborne illness in many regions, generating significant economic burdens due to medical expenses, work absences, and hospital resources [1, 5].

*Salmonella* infections occur worldwide and generally present as self-limiting gastroenteritis, characterized by diarrhea, fever, and dehydration [2, 3, 9]. The infectious dose for healthy adults ranges from 10³ to 10⁹ CFU/g, with an increased risk of severe outcomes in vulnerable populations, such as elderly individuals and cancer patients, in whom infection may progress to bacteremia or systemic disease [4, 7]. Disease severity is influenced by both strain virulence and host immune status [6, 9]. Although most cases resolve without antibiotic therapy, the growing emergence of antimicrobial resistance has complicated clinical management [2, 10, 11]. In Brazil, thousands of *Salmonella* associated gastroenteritis cases are reported annually; however, underreporting remains a concern, as surveillance systems often capture only severe outbreaks [8].

Transmission occurs primarily via the fecal–oral route through contaminated food, particularly animal-derived products such as poultry and pork, which account for the majority of foodborne salmonellosis cases [3, 4, 7, 12, 13]. *Salmonella* can persist in raw meat, irrigation water, agricultural products, and food-processing surfaces, facilitating cross-contamination throughout the production chain [3, 4, 7]. Among the circulating serovars, *Salmonella enterica* subsp. *enterica* serovar Typhimurium (*S. typhimurium*) is one of the most frequently isolated in foodborne outbreaks and is associated with both gastroenteritis and systemic infections in humans [6, 14]. This serovar is predominantly linked to pork and poultry production systems, with the monophasic variant being particularly prevalent in swine and pork products, underscoring the role of animal reservoirs and processing environments in transmission dynamics [6, 15–17].

Given the persistence of *S. typhimurium* in food production systems and its role in foodborne outbreaks, effective control measures are critical. *S. typhimurium* can survive under various conditions, persist in food-processing environments, and resist conventional disinfection methods [18–20]. To mitigate bacterial contamination in food, the industry relies on chemical preservatives and disinfectants; however, concerns regarding their toxicity and the emergence of antimicrobial resistance (AMR) have driven interest in alternative natural antimicrobial compounds, such as essential oils (EOs), offering promising alternatives for reducing microbial contamination and improving food safety [21, 22].

EOs are complex mixtures of bioactive plant secondary metabolites whose composition varies according to factors such as climate, plant age, and extraction method [23, 24]. EOs have attracted considerable attention for their antimicrobial, antioxidant, and preservative properties, with applications in food safety progressing from traditional in vitro assays to promising in situ studies conducted directly in food matrices [25–28]. Among them, Eucalyptus globulus essential oil (EGEO) and Cymbopogon flexuosus essential oil (CFEO) have demonstrated antimicrobial activity against foodborne pathogens, including *Salmonella* spp. [29, 30]; however, their mechanisms of action and metabolic effects under realistic food conditions remain insufficiently explored. Despite their natural origin, EO constituents may exert dose-dependent cytotoxic effects due to their lipophilic nature and capacity to interact with biological membranes [31]. Therefore, safety assessment using biologically relevant cellular models is essential. In this context, erythrocytes serve as sensitive indicators of membrane-disruptive and potential systemic toxicity, while hepatocyte-derived systems, such as zebrafish liver (ZF-L) cells, are particularly relevant given the central role of the liver in xenobiotic metabolism following ingestion. Together, these complementary models allow evaluation of membrane integrity and metabolic toxicity, contributing to a more comprehensive safety assessment for potential food applications [31, 32].

This study aims to evaluate the efficacy of commercial EGEO and *Cymbopogon flexuosus* CFEO extracted from leaves in controlling *S. typhimurium* contamination, assessing their antimicrobial activity and cytotoxic effects in both in vitro and in situ conditions, providing valuable insights into how these EOs influence *S. typhimurium* in a food-relevant setting. Additionally, the cytotoxicity of EGEO and CFEO was evaluated in erythrocytes and ZF-L cells, to assess their potential safety for food applications. By integrating antimicrobial and metabolic assessments with cytotoxicity evaluations, this study aims to uncover new perspectives on the application of EOs as natural preservatives and their potential role in controlling *S. typhimurium* contamination in the food industry.

## Materials and methods

### Reagents

*Eucalyptus globulus* essential oil (EGEO), was sourced from Ferquima (Brazil), and was extracted through steam distillation of the green leaves. Its primary components are eucalyptol, alpha-pinene, and gamma-terpinene. *Cymbopogon flexuosus* essential oil (CFEO), obtained from ViaAroma, is extracted through distillation of the fresh cut grass and contains major components such as citral, citronellol, geraniol, d-limonene, and linalool. Both oils are intended for professional and industrial use. Our research group previously analyzed the chemical composition of both EOs in another study (unpublished data).

Leibovitz’s L-15, Ham’s F-12, and high-glucose DMEM media were acquired from Vitrocell Embriolife (Brazil), and defibrinated sheep blood was obtained from Laborclin (Brazil). All chemical reagents used in this study were of analytical or pharmaceutical grade.

### Cytotoxicity assays

#### Hemolysis assay (HA)

The assay followed the protocol of Cerveira et al. (2021) [33] using defibrinated sheep blood (Laborclin, Brazil). Blood was centrifuged (1500 RPM, 10 min), plasma was discarded, and erythrocytes were resuspended in PBS (4% v/v). EGEO and CFEO were solubilized in DMSO (50% v/v), and 25 µL of this solution was diluted in PBS (100 µL), obtaining an initial DMSO concentration of 6.5%, followed by serial dilutions down to 0.001%. Triton X-100 (Neon, Brazil) was the positive control, and PBS was the negative control. Samples were incubated (37 °C, 30 RPM, 1 h) and centrifuged (800 × g, 10 min). The supernatant was transferred to a 96-well plate (KASVI, Brazil) and read at 405 and 425 nm in a UV-Vis spectrophotometer (Rosys Anthos, Switzerland).

#### ZF-L cells and initial processes

Cell viability was assessed using the MTT assay, which measures mitochondrial metabolic activity through the reduction of 3-(4,5-dimethylthiazol-2-yl)-2,5-diphenyltetrazolium bromide to formazan crystals, and the neutral red (NR) uptake assay, which evaluates lysosomal integrity based on the incorporation of the dye into viable cells. For these assays, zebrafish (*Danio rerio*) hepatocytes of the ZF-L cell line (Code 0256) were obtained from the Rio de Janeiro Cell Bank (BCRJ). Cells were cultured in a medium containing 50% Leibovitz’s L-15, 35% high-glucose DMEM, 10% Ham’s F-12, and 5% RPMI 1640, supplemented with NaHCO₃ (0.15 g/L), HEPES (15 mmol/L), insulin (0.01 mg/mL), 5% heat-inactivated fetal bovine serum (FBS), and antibiotics. Cultures were maintained at 28 °C without CO₂.

After 24 h of incubation (28 °C), treatments were applied in triplicate. EGEO and CFEO were first solubilized in dymethylsulfoxide (DMSO) and diluted in PBS to final concentrations ranging from 0.5% to 0.001% (nine serial dilutions), ensuring DMSO did not exceed 0.5% in the medium. Hydrogen peroxide (5% v/v) was the positive control, and PBS was the negative control. Plates were incubated (28 °C, 24 h) under mild agitation (30 RPM).

#### MTT assay

After overnight incubation, the medium from each well was collected for future analysis. An MTT solution (1 mg/mL, Invitrogen, USA) in PBS was prepared, and 100 µL was added per well. The plate was incubated at 28 °C for 4 h in the dark. Then, the medium was discarded, and 100 µL of absolute ethanol (Merck, Germany) was added to each well. After overnight incubation (27 °C, under gentle agitation), absorbance was measured at 570 nm using a microplate reader (Biochrom Anthos). This is an adapted protocol of Mossmann (1983) [34].

MTT is reduced by mitochondrial dehydrogenases in viable cells, forming insoluble purple formazan crystals. The intensity of the color indicates cell viability, with lower toxicity of the tested compound. Ethanol solubilizes the crystals, allowing spectrophotometric quantification [35].

#### NR uptake assay

After overnight incubation, the plate medium was discarded, and 100 µL of a NR (Invitrogen, USA)n solution at 40 µg/mL was added to each well. The plate was sealed and incubated for 4 h at 28 °C, protected from light. Then, the NR medium was discarded, and 100 µL of a bleaching solution composed of 50% absolute ethanol (Merck, Germany), 49% deionized water, and 1% glacial acetic acid (Merck, Germany) was added to each well. The plate was incubated overnight and read in a microplate reader (Rosys Anthos, Switzerland) at 570 nm. This protocol was adapted based on the findings of Borenfreund & Puerner (1985) [36].

NR is a weakly cationic dye at physiological pH, soluble in water, and can passively diffuse through cell membranes. In viable cells, NR accumulates in lysosomes due to electrostatic binding, as a proton gradient maintains a lower pH inside lysosomes. In cells with compromised lysosomal membranes, this gradient is lost, leading to NR deprotonation and release from lysosomes. The acidified alcoholic solution extracts NR from cells, allowing quantification by UV-Vis spectrophotometry [37].

### Antimicrobial behavior of EGEO and CFEO and bacterial susceptibility

EGEO and CFEO had their antimicrobial susceptibility against *S. typhimurium* tested through the Minimum Inhibitory and Bactericidal Concentration assays (MIC and MBC, respectively). After the establishment of these concentrations of each compound, time-kill assays were conducted to evaluate the antimicrobial behavior of EOs when influenced by time.

To perform such tests, one strain of *S. typhimurium* was selected from the available laboratory bacterial library. The strain is cataloged in the American Type Culture Collection (ATCC) under the code ATCC 14,028 and was isolated from the heart and liver tissues of 4-week old chickens and suitable for studies like ours [38].

The strain was cultured in first in nutrient broth and then in the selective agar Salmonella-Shigella (SS) (Kasvi, Brazil) and incubated at 37 °C for 24 to 48 h. For MIC, MBC and time-kill assays, bacteria were then inoculated at the 0.5 McFarland turbidity scale (1.5 × 10⁸ CFU/ mL) in a 0.9% (m/v) sodium chloride (NaCl) (Merck, Germany) solution.

#### MIC and MBC

The MIC was determined through the microdilution method, following the guidelines outlined in the M7-A6 protocol by the Clinical and Laboratory Standards Institute (CLSI) [39]. he experiment was performed using 96-well microplates, with each well containing 100 µL of Mueller Hinton (MH) broth (Kasvi, Brazil). EGEO and CFEO were subjected to serial dilution, ensuring a final well volume of 100 µL.

A 10 µL aliquot of the bacterial inoculum was introduced into each well. Control groups included a bacterial growth control, consisting of MH broth with the bacterial suspension, and a sterility control, containing only MH broth. The plates were incubated in an incubator (Láctea, Brazil) at 37 °C for 24 h under aerobic conditions. Bacterial growth was assessed visually based on turbidity, where increased turbidity indicated proliferation. The MIC was identified as the lowest concentration of each EO at which no visible bacterial growth was observed.

Following MIC determination, it was necessary to establish whether this concentration exerted bacteriostatic or bactericidal effects. While bacteriostatic activity halts bacterial growth, bactericidal action leads to cell death. To assess this, wells corresponding to the MIC, a lower concentration (0.5× MIC), and a higher concentration (2× MIC) were streaked onto MH agar plates (Kasvi, Brazil). These plates were then incubated aerobically at 37 °C for 48 h. The lowest concentration at which no bacterial colonies developed on the agar was defined as the MBC under the given experimental conditions.

#### Time-kill assays

The time-kill assays were performed following a modified version of the protocol described by Tsuji (2007) [40]. Prior to the experiment, bacterial strains were grown on selective agar plates for 24 h. The impact of EOs was monitored over a 48-hour period. To begin, bacterial suspensions adjusted to 0.5 on the McFarland scale were inoculated into test tubes containing 1 mL of MH broth for *S. typhimurium*. These tubes were incubated at 37 °C with continuous agitation using a shaker (Láctea, Brazil) set to mild agitation (30 RPM).

The experiment included seven different tube conditions: a sterility control (SC), a bacterial growth control (BC), and test tubes containing EO concentrations at MIC, 2× MIC, and 4× MIC. Samples from each tube were collected at nine distinct time points (0, 6, 12, 18, 24, 30, 36, 42, and 48 h). At each time interval, quantitative plating was performed to determine colony-forming units (CFUs), allowing the bacterial population in each tube to be assessed over time.

### Metabolic influence of EGEO and CFEO: essential oil-modified triple sugar iron agar assay

The Essential Oil-Modified TSI Agar Assay (EO-TSI Assay) was specifically designed in this study, aiming to evaluate the antimicrobial effects of EGEO and CFEO on *S. typhimurium* ATCC 14,028, focusing on their possible effects on key bacterial metabolic processes such as sugar fermentation, hydrogen sulfide (H₂S) production, and overall growth viability.

Triple Sugar Iron (TSI) agar medium (KASVI, Brazil) was prepared according to standard protocols, containing glucose (0.1%), lactose (1%), sucrose (1%), peptones, sodium thiosulfate (Na_2_O_3_S_2_), ferrous sulfate (FeSO_4_), and phenol red as a pH indicator. Medium was prepared using distilled water and autoclaved (marca da autoclave) until further use. Stock solutions of EGEO and CFEO were prepared and diluted in DMSO (Merck, Germany) to concentrations corresponding to 4x MIC, 2x MIC, and MIC obtained for the ATCC 14,028 strain. The diluted EOs were then added to the liquid TSI medium, ensuring thorough mixing to achieve homogeneity. The modified TSI agar was dispensed into sterile test tubes (Bioclin, Brazil) and allowed to solidify in a slanted position to create the appropriate agar surface for subsequent inoculation.

Isolated colonies of *S. typhimurium* ATCC 14,028, grown on SS agar, were selected for inoculation. A sterile inoculating needle was used to stab the TSI agar bottom and streak the slanted surface. The inoculated tubes were incubated at 36 °C for 24 h under aerobic conditions. Controls included a bacterial control (BC) with unmodified TSI agar inoculated with the bacterial strain and a sterility control (SC) consisting of unmodified TSI agar without bacterial inoculation.

After incubation, the tubes were examined for changes in color, black precipitate formation, and the presence of gas production. The color change in the medium was used to assess sugar fermentation, with yellow indicating acid production and red indicating no fermentation. The formation of black FeS precipitates indicated hydrogen sulfide (H₂S) production. The results were compared across the different concentrations of EGEO and CFEO to determine their impact on the metabolic activity of *S. typhimurium*.

### In situ assays

An in situ experiment was conducted to evaluate the preservative effects of EGEO and CFEO in pork contaminated with *S. typhimurium*, simulating a practical scenario in which meat remains at room temperature before undergoing brining and subsequent refrigeration prior to consumption. Before experimental inoculation, the pork samples were microbiologically analyzed to confirm the absence of *Salmonella* spp.

#### Experimental design

Freshly slaughtered pork sirloin (sourced from a local butchery in Pelotas, Rio Grande do Sul, Brazil) was cubed into standardized 10 g portions, ensuring the absence of preservatives. Each portion was placed in a sterile Falcon tube, followed by the addition of 1 mL of a *S. typhimurium* (ATCC 14028) inoculum, prepared at a concentration of 10⁴ CFU/mL. The inoculum concentration was selected to simulate a hypothetical scenario in which ingestion could lead to infection. The inoculated meat samples were incubated at room temperature for 5 h to allow bacterial colonization. Subsequently, 10 mL of a pre-prepared brine solution was added to each tube, and the samples were stored under refrigeration (8 °C ± 2 °C) for seven days.

#### Brine preparation

A stock brine solution was prepared using distilled water supplemented with 1% (v/v) NaCl. The experiment was divided into four groups: (1) a control group containing brine without EOs; (2) an EGEO treatment group, where EGEO was added at 10× MIC for *S. typhimurium*; (3) a CFEO treatment group, with CFEO added at the same concentration (10× MIC); and (4) a sterility control group, consisting of 10 g of refrigerated meat without brine or bacterial inoculation, to confirm the absence of *Salmonella* prior to inoculation. Except for the sterility control, all groups received the bacterial inoculum. The 10× MIC concentration was selected because in situ experiments require higher compound concentrations due to interactions with proteins and lipids in the food matrix. The sterility control was considered only for microbiological analyses and visual assessment. All tests were performed in triplicate.

#### Subsequent analyses

Microbiological analyses were conducted on days 1 and 7 to assess the presence or absence of *Salmonella* spp. in the brined meat. These evaluations aimed to determine whether the EOs could enhance food safety by reducing or eliminating bacterial load and mitigating the risk of infection. Additionally, brine pH was measured on days 1, 3, and 7. At these same time points, electrolyte concentrations (Mg²⁺ and Ca²⁺) were analyzed to assess the preservative effects of the EOs. Photographs of the meat were taken on days 1, 3 and 7 to document visible changes in appearance.

#### Visual assessment of meat and microbiological analysis

At days 1, 3 and 7, refrigerated meat was photographed using an iPhone 11 (Apple, USA) for visual assessment in differences in color and overall integrity. At days 1 and 7, samples were subjected to microbiological isolation procedures of Salmonella spp. as instructed by the American Public Health Association [41]. The procedure began with a pre-enrichment step, in which the meat sample was homogenized with 225 mL of buffered peptone water. The mixture was then incubated at 37 °C for 24 h to allow bacterial growth.

Following pre-enrichment, an enrichment step was performed to enhance the detection of *Salmonella* spp. For this, 1 mL of the pre-enrichment broth was transferred to a tube containing 10 mL of tetrathionate broth (TTB, LabM, USA), while 0.1 mL was inoculated into a separate tube containing 10 mL of Rappaport-Vassiliadis broth (RVB, Accumedia, USA). The TTB was incubated at 35 °C, whereas the RVB was incubated at 42 °C in a water bath, both for 24 h. After enrichment, a loopful of each culture was streaked onto xylose lysine desoxycholate (XLD, Kasvi, Brazil) agar plates and Bismuth-sulphite (BSA, Himedia, India) agar to obtain isolated colonies. The inoculated plates were then incubated at 35 °C for 24 h. Identification of suspect Salmonella colonies was based on their characteristic appearance on selective media and biochemical testing on TSI, LIA and UREA (Kasvi, Brazil) agars.

The decision to perform presence/absence analysis was based on Brazilian microbiological standards established by the National Health Surveillance Agency (ANVISA), specifically Instrução Normativa nº 161/2022, which adopt a zero-tolerance criterion for *Salmonella spp*. in meat products. According to these guidelines, the detection of even a single viable cell in a defined sample unit is considered a potential public health risk, and therefore compliance is determined qualitatively rather than quantitatively.

#### pH measures

The hydrogen potential (pH) of the samples were measured using a digital pHmeter (MyLabor, Brazil), equipped with an ionic electrode and thermometer. The pH measurement was performed only in the brines at days, 1, 3 and 7 of the experiment and results were expressed as mean ± standard deviation (SD) of the triplicates.

#### Electrolytes concentrations

The release of magnesium (Mg²⁺) and calcium (Ca²⁺) ions from meat to brines was performed after refrigeration for 1, 3, and 7 days, as described by Ferrer et al. (2024) [42], with modifications. This test was conducted using a colorimetric assay with a monoreagent Mg kit (Bioclin^®^, Brazil). Absorbance at 500 nm was measured using a Cobas MIRA^®^ automated analyzer (Roche Diagnostics, Switzerland), following the manufacturer’s instructions. Mg²⁺ concentration was calculated using the following equation:

1 $$[\mathrm{M}\mathrm{g}^{2+}]=\left[\right({\mathrm{A}\mathrm{b}\mathrm{s}}_{\mathrm{s}\mathrm{a}\mathrm{m}\mathrm{p}\mathrm{l}\mathrm{e}}\times\:2)/{\mathrm{A}\mathrm{b}\mathrm{s}}_{\mathrm{s}\mathrm{t}\mathrm{a}\mathrm{n}\mathrm{d}\mathrm{a}\mathrm{r}\mathrm{d}}]$$

where Abs_sample_ is the absorbance of samples and Abs_standard_ is the absorbance of the standard reagent.

As the reaction follows Beer-Lambert’s Law, a calibration factor (CF) can be used:

$$\:\mathrm{C}\mathrm{F}=\left[\mathrm{S}\mathrm{t}\mathrm{a}\mathrm{n}\mathrm{d}\mathrm{a}\mathrm{r}\mathrm{d}\right]/\left[{\mathrm{A}\mathrm{b}\mathrm{s}}_{\mathrm{S}\mathrm{t}\mathrm{a}\mathrm{n}\mathrm{d}\mathrm{a}\mathrm{r}\mathrm{d}}\right],\:\mathrm{w}\mathrm{h}\mathrm{e}\mathrm{r}\mathrm{e}\:\left[\mathrm{S}\mathrm{t}\mathrm{a}\mathrm{n}\mathrm{d}\mathrm{a}\mathrm{r}\mathrm{d}\right]\:\mathrm{i}\mathrm{s}\:2\:\mathrm{m}\mathrm{g}/\mathrm{d}\mathrm{L}$$

The reaction principle uses the Mann and Yoe/Magon Sulfonate/ Xylidyl Blue dye. In an alkaline pH and in the presence of Mg, the dye develops a red color, the intensity of which is proportional to the Mg concentration. The method does not require protein precipitation, making it more sensitive than the Titan Yellow method.

Ca²⁺ concentration was measured using the end-point colorimetric method with Arsenazo III. Ca²⁺ reacts with Arsenazo III in an acidic medium, forming a blue-colored complex, the intensity of which is proportional to the Ca²⁺ concentration in the sample. The absorbance of the reaction product was measured using a Cobas MIRA^®^ automated analyzer, at a wavelength between 600 and 680 nm. Equation 2. was used to calculate Ca²⁺ concentrations.

2 $$[\mathrm{C}\mathrm{a}^{2+}]=\left[\right({\mathrm{A}\mathrm{b}\mathrm{s}}_{\mathrm{s}\mathrm{a}\mathrm{m}\mathrm{p}\mathrm{l}\mathrm{e}}\times\:10)/{\mathrm{A}\mathrm{b}\mathrm{s}}_{\mathrm{S}\mathrm{t}\mathrm{a}\mathrm{n}\mathrm{d}\mathrm{a}\mathrm{r}\mathrm{d}}]$$

All concentrations are expressed as mg/dL.

### Statistical analyses

Data analysis was performed using GraphPad Prism 8.0 (GraphPad Software, USA) for the HA, MTT, NR uptake assays, time-kill assays and pH, Mg²⁺ and Ca²⁺ measurements.

For cytotoxicity assays, data were expressed as the mean ± SD for triplicates using one-way analysis of variance (ANOVA) followed by Dunnett’s post-hoc test.

For the time-kill assays, statistical analysis was performed using the Mantel-Cox (Log-rank) test, the Gehan-Breslow-Wilcoxon test, and the Mantel-Haenszel test. These tests were applied to evaluate the differences in survival curves between experimental groups, accounting for the variation in bacterial count over time. The Mantel-Cox test was used to assess overall survival differences, while the Gehan-Breslow-Wilcoxon and Mantel-Haenszel tests were employed to focus on survival at different time points and to control for confounding variables. These tests were chosen for their ability to handle censored data and to compare the time-to-event distributions among the groups effectively.

pH, Mg²⁺ and Ca²⁺ concentration data were analyzed using a nonlinear regression model to assess trends over time. For comparisons across multiple time points and treatment conditions, a two-way ANOVA was conducted, followed by Tukey’s post-hoc test for multiple comparisons. Normality was assessed using the Shapiro-Wilk test., with statistical significance set at *p* < 0.05.

Microbiological analysis was represented in a heatmap using the software Flourish (United Kingdom).

## Results and discussion

### Cytotoxicity assays

Cytotoxicity assays were conducted using mammalian red blood cells and zebrafish hepatocytes to comprehensively evaluate the potential toxic effects of the tested compounds on both adherent and non-adherent eukaryotic cells. The selected assays— HA, MTT, and NR uptake— are well-established and widely used methods for assessing different aspects of cytotoxicity, and by employing this multifaceted approach, we could determine the cytotoxic behavior of the compounds and glimpse into possible mechanisms of action in eukaryotic cells.

**Fig. 1:**
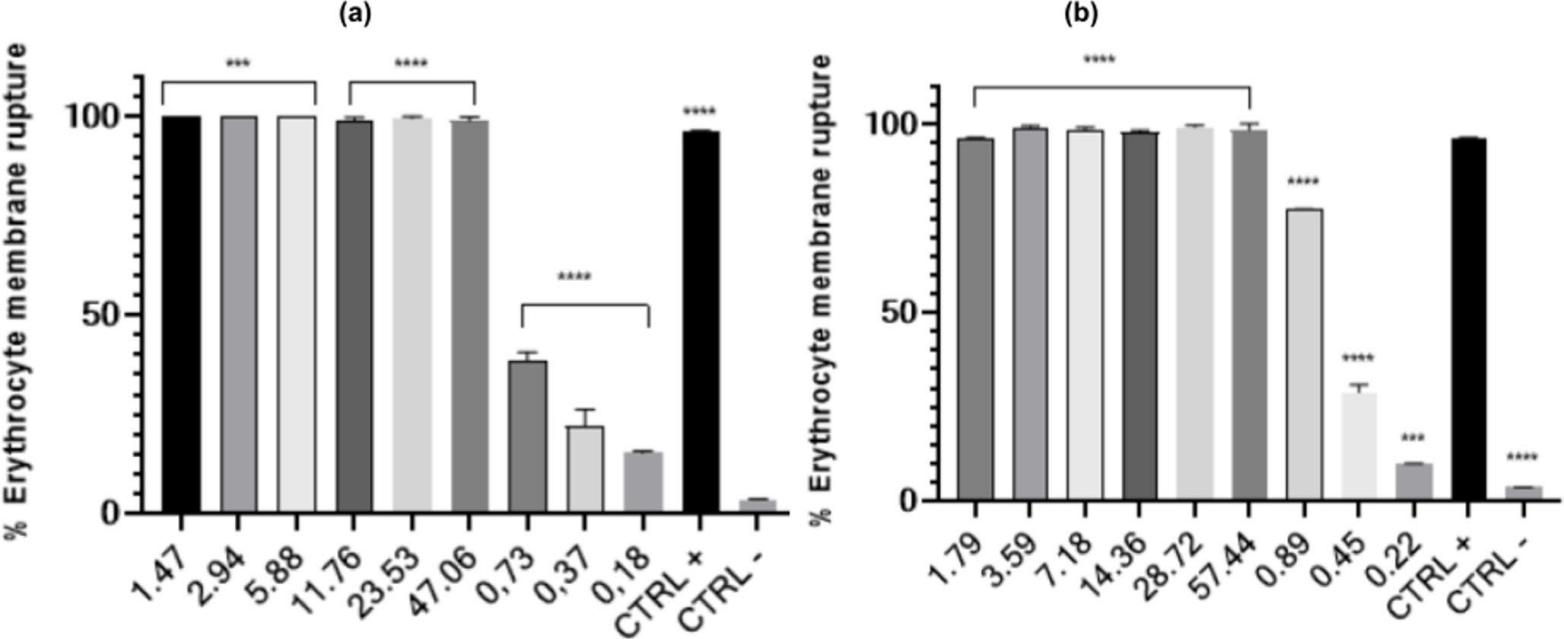
Hemolytic activity of CFEO (**a**) and EGEO (**b**). Values are represented as mean ± SD from duplicates. * *p* < 0.05, ** *p* < 0.01, *** *p* < 0.001, **** *p* < 0.0001. Compared to the positive control (CTRL +)

In the HA, both CFEO and EGEO caused erythrocyte rupture at all tested concentrations, with hemolytic activity being similar between the compounds. This assay was used as a preliminary screening method to determine appropriate concentration ranges before applying the compounds to ZF-L hepatocytes for cytotoxicity testing. The tested concentration range (6.25 − 0.001% v/v) exhibited high cytotoxicity in erythrocytes up to 0.5% (v/v). A lower concentration range (0.5–0.001% v/v) exhibited significantly less hemolytic activity. This lower range was selected to ensure the feasibility of subsequent cell viability assays. The results of the hemolytic activity are presented in Fig. 1.

Hemolytic activity of CFEO can cause alterations in erythrocyte membranes at high concentrations [43], which aligns with our findings. Other techniques, such as microencapsulation of CFEO, reduced its hemolytic activity while increasing its anti-acetylcholinesterase activity, suggesting its potential as a formulation strategy for pharmaceuticals and preservatives containing this EO [44].

Godoi et al. (2017) [45] compared the hemolytic activity of free EGEO and its nanoemulsified forms. Their results indicated that formulation safety was compromised at higher EGEO concentrations, as increased hemolytic activity was observed due to erythrocyte membrane rupture caused by eucalyptol. However, low concentrations (0.2% EGEO, v/v) did not cause hemolysis, which also aligns with our results.

**Fig. 2:**
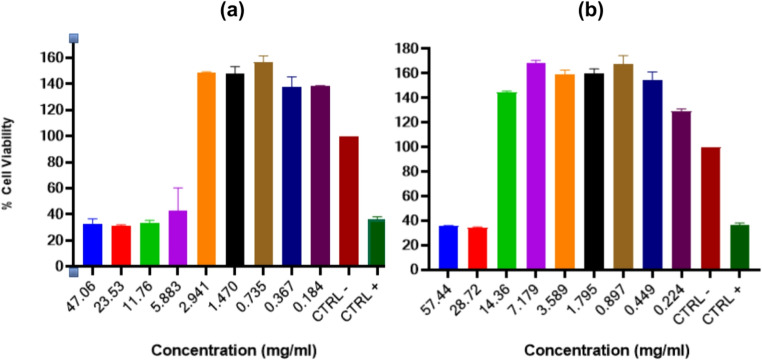
Cell viability assessed by the MTT assay after treatment with **a**) CFEO and (**b**) EGEO. Values are represented as mean ± SD from triplicates. * *p* < 0.05, ** *p* < 0.01, *** *p* < 0.001, **** *p* < 0.0001. Compared to the negative control (CTRL -)

The MTT assay allowed us to observe the maintenance of cell viability after treatment with CFEO from the fifth dilution (2.94 mg/mL) and with EGEO from the third dilution (14.3 mg/mL). Therefore, it can be stated that EGEO is less cytotoxic to hepatocytes than CFEO, as the cells did not undergo cell death even at higher concentrations. The results were compared with the positive control used (H₂O₂). A cell viability above 100% was obtained for both oils, indicating that, at these concentrations, the compounds were not cytotoxic and did not impede cell proliferation. The results are presented in Fig. 2.

**Fig. 3:**
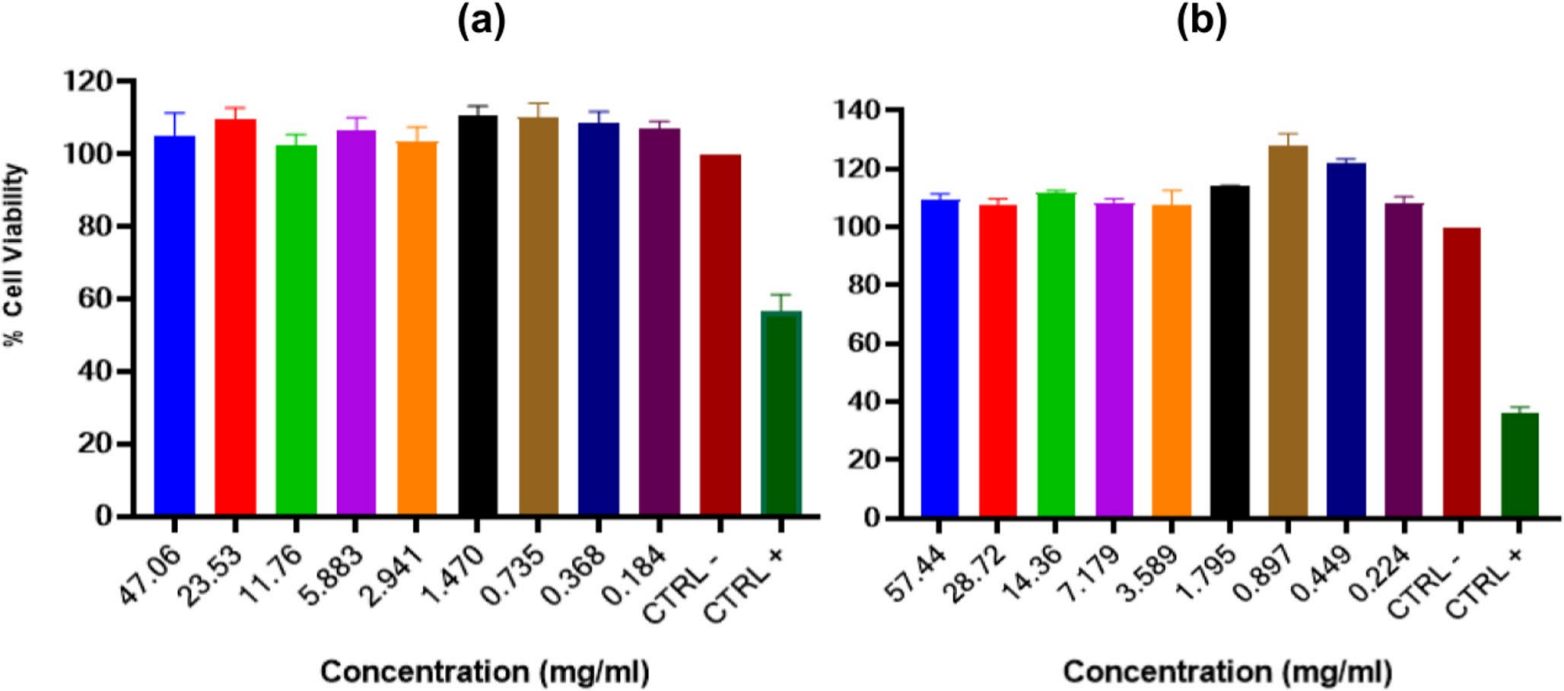
Cell viability assessed by the NR uptake assay after treatment with **a**) CFEO and (**b**) EGEO. Values are represented as mean ± SD from triplicates. * *p* < 0.05, ** *p* < 0.01, *** *p* < 0.001, **** *p* < 0.0001. Compared to the negative control (CTRL -)

Regarding NR uptake, the compounds did not exhibit cytotoxic activity from the first tested concentration. This indicates that the compounds are not capable of altering lysosomal function at the tested concentrations. The results are presented in Fig. 3.

The high concentration of eucalyptol, as determined by gas chromatography analysis (data not shown) in the tested EGEO, may explain the high cell viability observed. This is because eucalyptol has a low ability to penetrate eukaryotic cell membranes, being primarily transported by carrier proteins [46]. Eucalyptol is commonly used as a flavoring agent in products such as toothpaste, syrups, and mouthwashes, partly due to its low cytotoxicity.

The tested EGEO also contained high concentrations of low-cytotoxicity compounds such as α-pinene [47]. Adukwu et al. (2016) [48] demonstrated that CFEO did not exhibit cytotoxicity at concentrations below 0.25% (v/v) in human dermal fibroblasts (HDF). However, the major compound in CFEO, citral, showed better biological activity, with viability reaching 80% when citral concentration reached 0.03% in the tested cells. A similar result was obtained in this study, where cell viability was higher at CFEO concentrations below 0.25% in ZF-L cells.

Other studies suggest a directly proportional relationship between citral and geraniol concentrations in CFEO and increased cytotoxicity in eukaryotic cells, indicating a synergistic effect with minor compounds that may reduce CFEO cytotoxicity. An alternative approach is the use of formulations such as nanoemulsions and gas-phase CFEO to reduce cytotoxicity and enhance stability in mixtures with volatile compounds [43, 45].

It is important to emphasize that the interpretations presented here are explanatory hypotheses derived from literature-supported reasoning and do not represent direct conclusions from our experimental data. Our research group intends to investigate these proposed mechanisms experimentally in the near future.

### Antimicrobial behavior of EGEO and CFEO

The results presented in Table 1 show the MIC and MBC of CFEO and EGEO against *S. typhimurium* (ATCC 14028). CFEO demonstrated a significantly lower MIC and MBC value (0.37 mg/mL), indicating a higher antimicrobial potency compared to EGEO, which required a MIC and MBC of 1.79 mg/mL to achieve similar effects. In both EOs the MIC was also bactericidal in the specific followed protocol.

**Table 1:** MIC/MBC values of tested EOs against *S. typhimurium* (ATCC 14026)

Bacteria	CFEO		EGEO	
	MIC	MBC	MIC	MBC
*S. typhimurium*	0,37	0,37	1,79	1,79

(-) MBC values were not present in the tested concentration range. All tests were performed in triplicates.

These findings suggest that CFEO is more effective in inhibiting bacterial growth and inducing cell death at lower concentrations, which may be attributed to its specific chemical composition and mechanisms of action. The equal MIC and MBC values for both EOs indicate that their bacteriostatic and bactericidal effects occur at the same concentration, initially suggesting a direct killing effect rather than merely growth inhibition. Plants of the *Cymbopogon* genus are known for its low MIC values, specially because the EOs of this genus are said to be good membrane disruptors [49]. Other studies indicated high MIC/MBC values for clinical isolates and other serovars of *Salmonella*, which does not align with ours [50]. There are few studies that access the *Cymbopogon flexuosus* specifically.

Additionally, the absence of MBC values outside the tested concentration range confirms that the selected concentrations were sufficient to determine the bactericidal potential of both EOs and that the strain is sensitive to these compounds. These results highlight CFEO as a more potent candidate for antimicrobial applications against *S. typhimurium*, while EGEO, despite requiring higher concentrations, still exhibits notable antimicrobial activity.

Comparing with the citotoxicity assays, CFEO at MIC/MBC (0,37 mg/mL) was not considered a particularly hemolytic concentration, and did not impair mitochondrial and lysossomal functions in ZF-L cells. Similarly, EGEO at MIC/MBC (1,79 mg/mL) was not cytotoxic in the MTT and NR uptake assays, but it is a high hemolytic concentration.

**Fig. 4:**
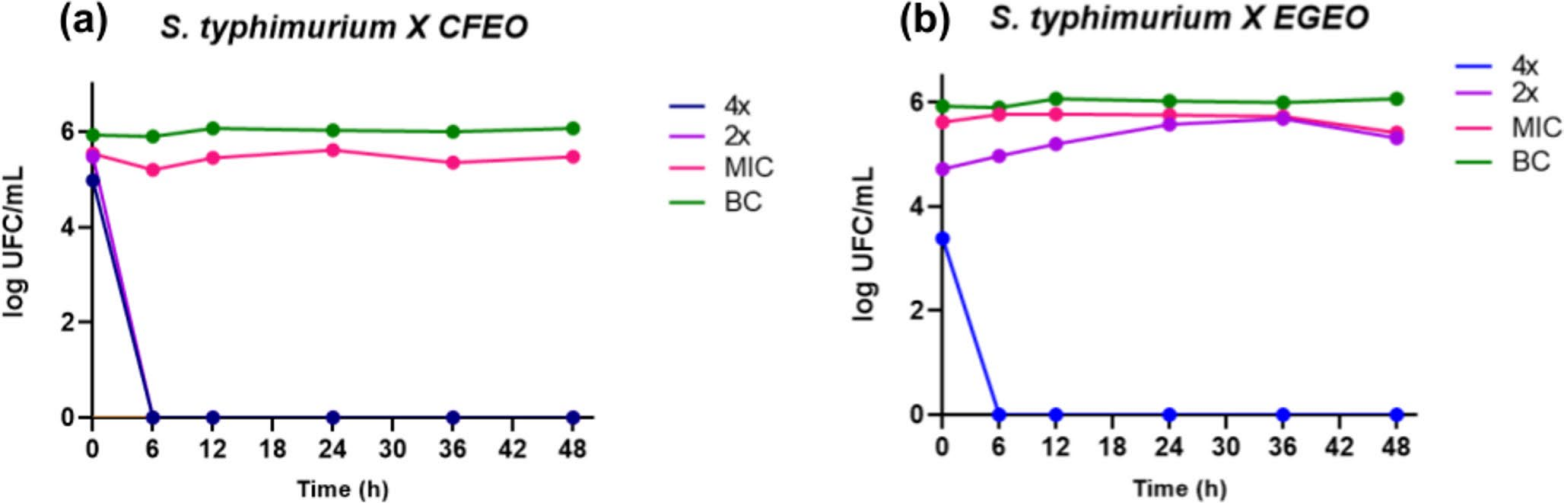
Bacterial killing curve of EGEO and CFEO in 4x, 2x and MIC concentrations against *S. typhimurium* (ATCC 14026). (**a**) killing curve of *S. typhimurium* treated with EGEO and (**b**) killing curve of *S. typhimurium* treated with CFEO. Treatments were applied at 4x, 2x and MIC concentrations. Data are expressed as curves representing the log (CFU*1000) of colonies counted by quantitative seeding. BC: bacterial control

To further investigate the antimicrobial behavior of these EOs over time and under constant agitation, the time-kill assays were employed. Results are shown in Fig. 4.

The time-kill assay results demonstrate that CFEO exhibits a potent bactericidal effect within six hours at concentrations of 4× and 2× MIC, as evidenced by the complete elimination of viable colonies. At MIC concentration, CFEO displayed only an inhibitory effect, with a noticeable reduction in colony counts compared to the bacterial control (BC), suggesting a dose-dependent antimicrobial response. Similarly, EGEO at 4× MIC also exhibited bactericidal activity within six hours, whereas lower concentrations (2× MIC and MIC) only inhibited bacterial growth, with reduced colony counts relative to the BC. CFEO showed similar results in time-kill assays against *E. coli* and *S. aureus* having complete bactericidal effect in 6 h, which was also verified for *S. tyhpimurium* [51, 52]. A study using CFEO at 0,25 x its MIC concentration for E. coli also showed that it starts to reduce bacterial counts at 6 h [53]. Studies with different serovars of *Salmonella* indicate that the genus seem quite resilient to EGEO composition and had significant higher MIC and MBC values that those found in our study [54, 55].

The observed differences in bactericidal efficacy can be attributed to the distinct chemical compositions of CFEO and EGEO. CFEO is strongly associated with membrane disruption [48, 49], which likely accounts for its rapid and pronounced bactericidal activity, making it a promising candidate for disinfection purposes. In contrast, while EGEO appears less effective in compromising bacterial membranes [46], its inhibitory effects suggest an alternative mode of action, possibly targeting bacterial metabolism rather than direct membrane disruption. This indicates that EGEO could have valuable applications beyond direct bactericidal activity, particularly in modulating bacterial metabolic processes, which could be explored for novel antimicrobial strategies.

Overall, the time-kill data reinforce the potential of CFEO as a rapid-acting bactericidal agent, while EGEO’s inhibitory effects warrant further investigation to elucidate its precise mechanism of action and potential applications in bacterial control.

### EO-TSI modified agar assay

**Fig. 5:**
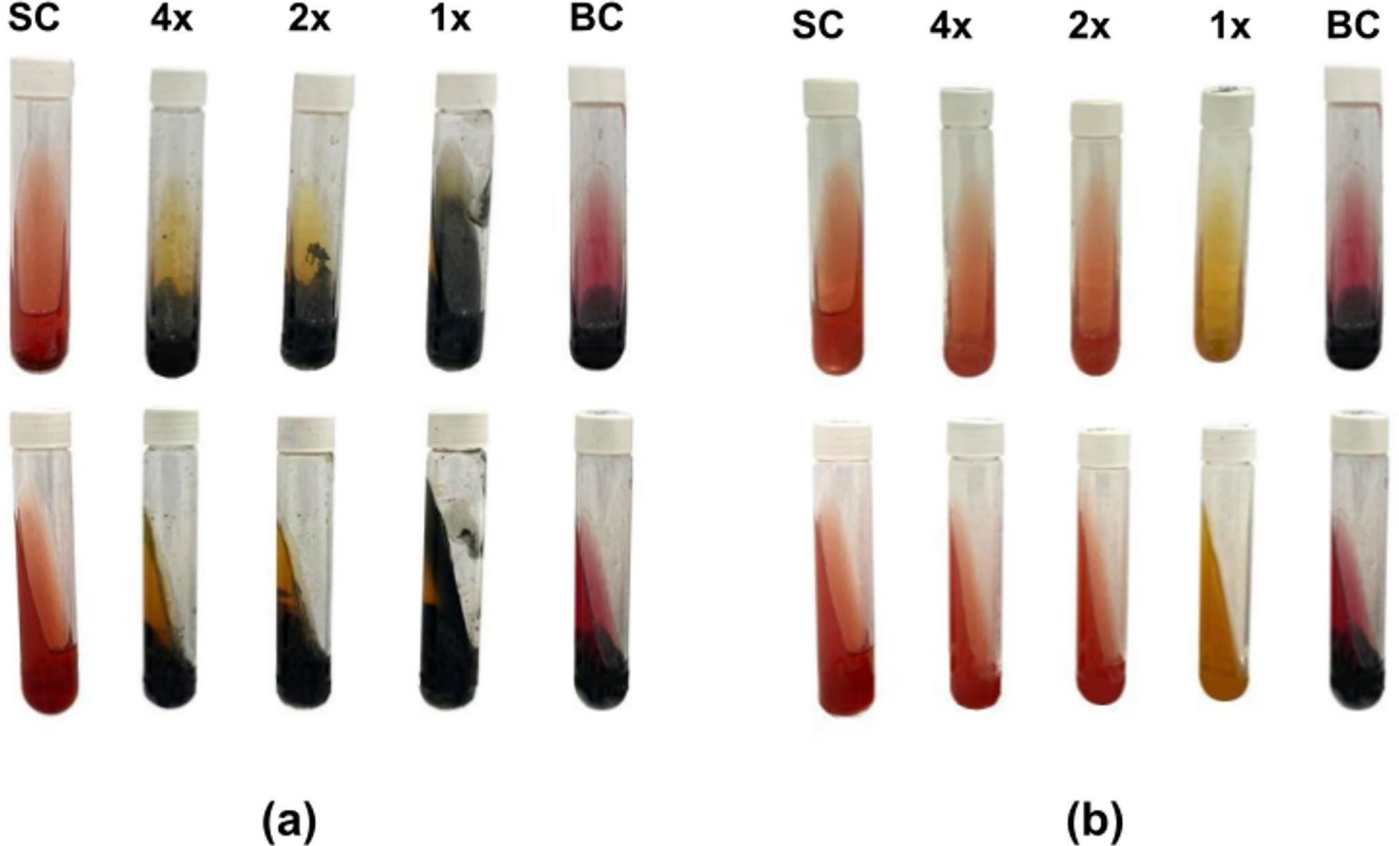
EO-TSI Modified Agar Assay. **a** Effects of *Cymbopogon flexuosus* essential oil (CFEO) and (**b**) *Eucalyptus globulus* in *S. typhimurium* (ATCC 14026). SC: sterility control; BC: bacterial control

The Triple Sugar Iron (TSI) medium is a widely used differential medium for the identification of Gram-negative enteric bacteria, such as *Salmonella* and *Escherichia coli* [56]. In this study, we adapted the conventional TSI test methodology to qualitatively assess the impact of EGEO and CFEO on microbial metabolism by incorporating the essential oils directly into the TSI medium. This approach enabled the evaluation of their effects on a model enteric bacterium, object of this study, *S. typhimurium*, with a focus on identifying key biochemical alterations in response to different EO concentrations. The visual results are presented in Fig. 5, while Table 2 summarizes the major metabolic changes observed.

**Table 2:** Effect of the *Eucalyptus globulus* essential oil (EGEO) and *Cymbopogon flexuosus* (CFEO) on *S. typhimurium* ATCC 14,026 metabolism

Compound	Concentration	Color	H₂S Production	Visible colonies (slant)
EGEO	4X MIC	Red	None	None
	2X MIC	Red	None	None
	1X MIC	Yellow	None	None
CFEO	4X MIC	Pink*	Present*	Present
	2X MIC	Yellow	Present**	Present
	1X MIC	Yellow	Present**	Present
BAC CTRL	-	Yellow	Present**	Present
ST CTRL	-	Red	None	None

N/N: red coloration, no change occurred. A/A: yellow coloration, acidification of the medium in both the base and the surface. K/A: pink coloration on the surface and yellow in the base, alkalinization of the surface and acidification of the base. *: only slant part of test tube; **: only bottom part of test tube

Analyzing the results from Fig. 5, it is observed that SC confirmed the absence of contamination throughout the experiment. The *S. typhimurium* BC presented the expected results: a K/A tube, H₂S production, and gas displacement (even if not visibly apparent), confirming the viability of the strain used.

Regarding the results for CFEO, the presence of the oil in the medium did not inhibit glucose fermentation or H₂S production. However, the oil prevented the oxidative degradation of peptones, which are used as an energy source by the bacterium, resulting in A/A tubes with black precipitates.

For EGEO, the 4x and 2x MIC concentrations did not cause any color changes, remaining similar to SC. Two hypotheses were proposed to explain this behavior: (1) the immediate death of the microorganisms, resulting in tubes with no microbial activity, or (2) a significant reduction in bacterial metabolism, to the point of blocking glucose fermentation and the consequent acidification of the medium. (1) was ruled out after streaking samples from the first two tubes, in which bacterial growth was observed. At the 1x MIC concentration, EGEO did not prevent glucose fermentation but inhibited peptone oxidation. The most intriguing finding was that, at all EGEO concentrations, no H₂S production was observed showing a strong impact in sulfur metabolism.

These results provide important insights, such as the potential interference of EGEO directly in the bacterium’s sulfur metabolism, blocking H₂S production even at concentrations that still allow glucose fermentation. This suggests a targeted action that may indicate novel antimicrobial mechanisms beyond simple cell destruction. In the case of CFEO, it is evident that neither sulfur metabolism nor glucose fermentation was affected, and bacterial proliferation occurred. However, there was a direct influence on peptone oxidation.

The correlation between MIC/MBC, time-kill, and EO-TSI results provides a comprehensive understanding of the antimicrobial and metabolic effects of EGEO and CFEO on *S. typhimurium*. This metabolic disruption could significantly impact *S. typhimurium* virulence, as sulfur metabolism is essential for multiple cellular functions, including energy production, stress response, and biofilm formation [57–60]. Inhibiting H₂S synthesis may reduce the bacterium’s ability to modulate host immune responses and resist oxidative stress, potentially weakening its pathogenicity [58, 60]. Meanwhile, the inhibition of peptone oxidation by CFEO could impair nitrogen assimilation, further restricting bacterial growth and survival in nutrient-limited environments [61]. These findings highlight not only the direct antimicrobial effects of EGEO and CFEO but also their ability to alter key metabolic pathways, which may influence bacterial fitness and virulence in host environments.

### In situ analyses

#### Visual assesment and microbiological analyses

**Fig. 6:**
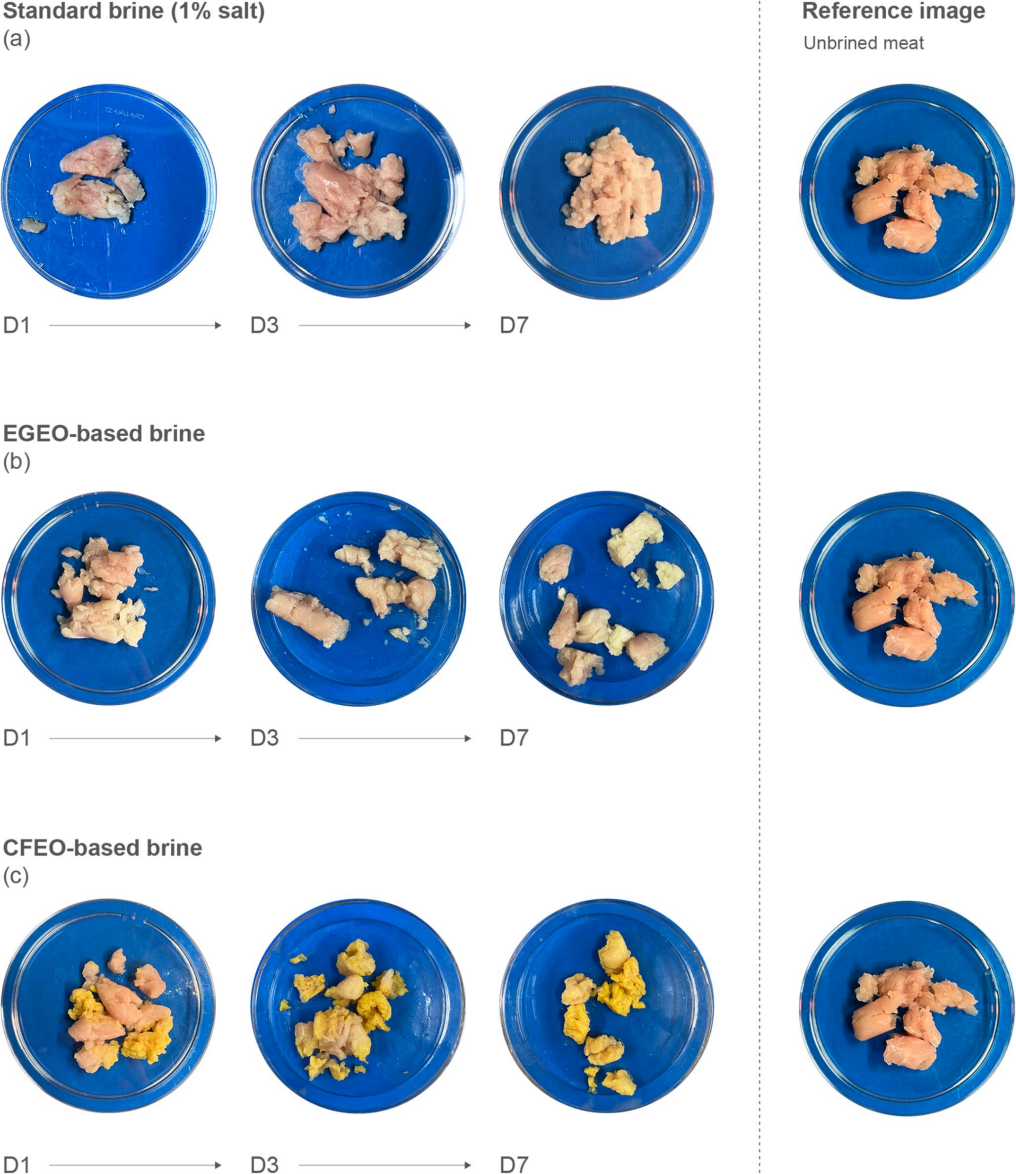
Visual assessment of effects of different brine formulations on meat appearance over time. Representative images of meat samples treated with (**a**) standard brine (1% salt), (**b**) EGEO-based brine, and (**c**) CFEO-based brine, observed at days 1 (D1), 3 (D3), and 7 (D7). A reference image of unbrined meat is shown for comparison

The brined portions of inoculated pork meat that underwent refrigeration for a week were microbiologically tested for presence or absence of *Salmonella spp.* at the beginning (day 1) and end (day 7) of the experiment. Photos were taken to assess the color and visual aspects of the brined meat portions and are represented in Fig. 6.

The CFEO-based brine treatment shows progressive discoloration and yellowing over time, while the EGEO-based brine preserves the meat’s structural integrity better but still shows some degradation. Both EO treatments exhibited a strong odor characteristic of the respective EO.

The standard brine maintains the meat’s original appearance with minimal changes, suggesting its effectiveness in preserving visual quality. Among the EO treatments, the EGEO-based brine most closely resembles the standard brine in terms of appearance. When compared to the reference image of unbrined meat, it is evident that the brining process, regardless of formulation, alters the meat’s appearance over time and influences the texture and color, with EOs potentially introducing additional preservative effects.

**Fig. 7:**
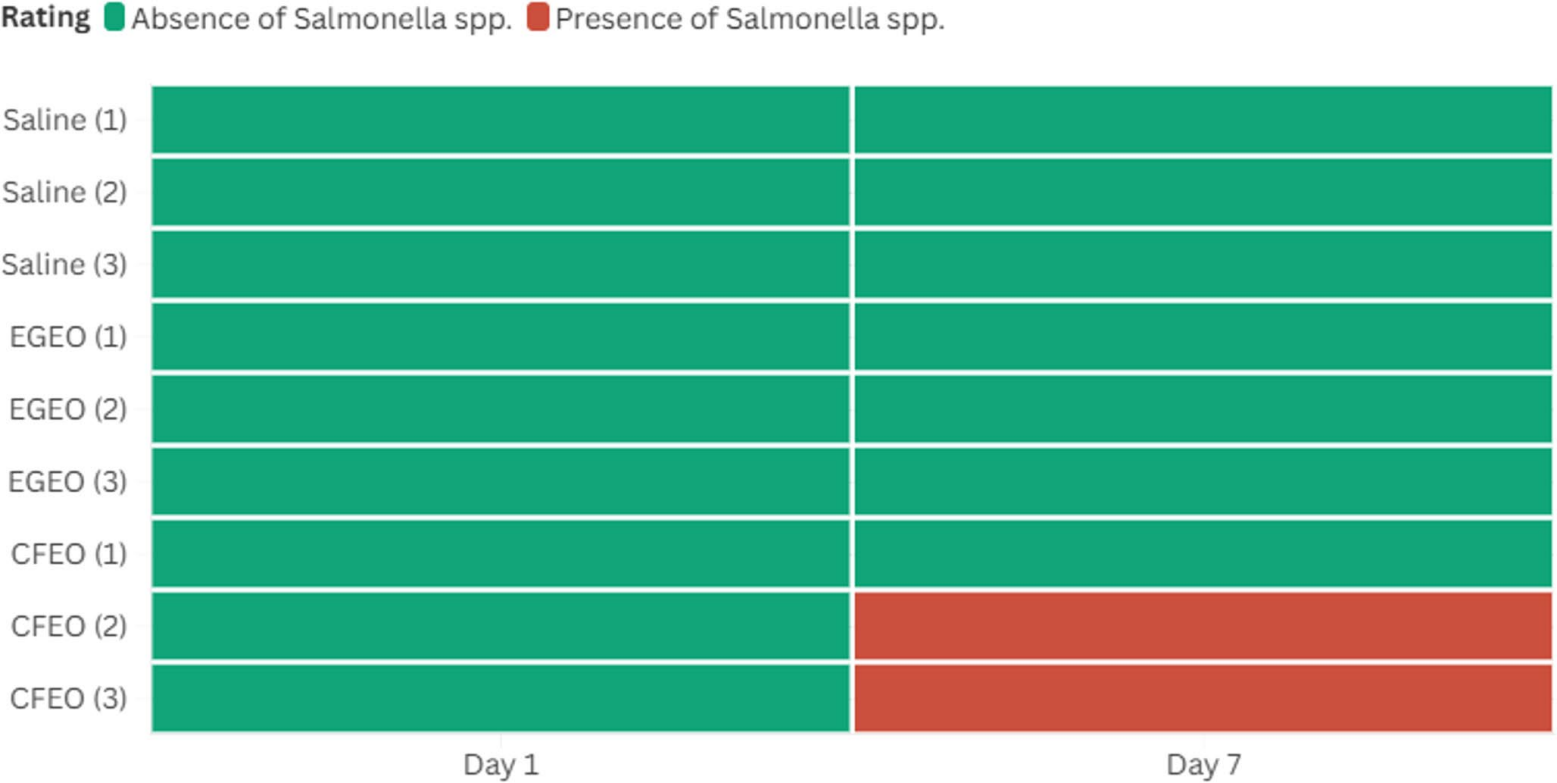
Heatmap of the microbiological analysis of *Salmonella* spp. presence in inoculated pork sirloin samples marinated in a standard brine (saline solution) and brines containing *Eucalyptus globulus* essential oil (EGEO) or *Cymbopogon flexuosus* essential oil (CFEO) at 10× MIC under refrigeration for 7 days. Results are presented for days 1 and 7. Green bars indicate the absence of *Salmonella* spp., while red bars denote its presence. Each treatment was performed in triplicate. Samples with suspected *Salmonella* colonies underwent biochemical confirmations. Heatmap generated using Flourish software

The microbiological analyses show that *Salmonella* spp. was absent in all samples on day 1, regardless of the treatment applied. However, on day 7, *Salmonella* spp. was detected in two of the three replicates for the CFEO-treated group, while the saline and EGEO groups maintained total bacterial inhibition. The results of the microbiological analyses are represented in Fig. 7.

These results suggests that CFEO was less effective than EGEO in preventing the survival of *Salmonella* spp. over the storage period. One possible explanation is that the antimicrobial compounds in CFEO, while initially effective, may have undergone degradation or diffusion over time, reducing their potency. In contrast, EGEO appears to have sustained its antimicrobial activity throughout the experiment. The complete absence of *Salmonella spp.* in the control saline group could be attributed to the natural inhibitory effects of the refrigeration process, combined with the initial bacterial inoculum. However, this does not exclude the possibility that *Salmonella* levels may increase over a longer storage period if no antimicrobial intervention is applied.

#### Brine analyses

The brines had their pH, and Mg²⁺ and Ca²⁺ levels assessed on days 1, 3 and 7. Regarding pH measurements, values are summarized in Table 3.

**Table 3:** pH values (Mean ± SD) in inoculated pork sirloin samples stored in different brines over time

Time (Days)	Saline Solution	EGEO	CFEO
1	5.62 ± 0.34	5.20 ± 0.62	3.86 ± 0.70
3	5.96 ± 0.11	6.07 ± 0.17	6.07 ± 0.17
7	5.93 ± 0.10	6.02 ± 0.09	6.00 ± 0.09

Hydrogen potential (pH) values in refrigerated contaminated pork meat kept in different brines over time. Table Z shows the pH variations in pork meat samples immersed in three different brine solutions: a control brine (saline solution), a brine containing *Eucalyptus globulus* essential oil (EGEO), and a brine containing *Cymbopogon flexuosus* essential oil (CFEO). Measurements were taken at three time points (1, 3, and 7 days). Data are presented as mean ± standard deviation (SD) from three independent replicates

The initial pH values of the samples displayed noticeable differences between treatments, with CFEO brine exhibiting a significantly lower pH (3.86 ± 0.70) compared to the brine control (5.62 ± 0.34) and EGEO (5.20 ± 0.62) on day 1. This suggests that CFEO may have contributed to an increased acidity in the initial phase of storage. However, from day 3 onwards, all samples reached similar pH levels, indicating a potential buffering effect of the meat or a gradual neutralization process.

By day 7, all treatments stabilized around a pH of 6.00, with minor variations between groups. The gradual increase in pH over time in the CFEO group suggests that its initial acidifying effect was transient, likely due to interactions between EO components and the meat matrix. These pH fluctuations may influence bacterial growth, enzymatic activity, and meat preservation, as lower pH values are typically associated with antimicrobial effects [63], while a shift toward neutral pH may promote spoilage-related microbial activity. Further microbiological analysis is required to determine whether these pH variations correlate with bacterial load differences.

In contrast, poorly preserved meat typically exhibits a decrease in pH due to the accumulation of acidic byproducts (e.g., lactate) and the activity of endogenous enzymes, which drive cellular degradation [63, 64]. However, prolonged high acidity could indicate autolysis, a process that is effectively inhibited by refrigeration [64–66]. Additionally, refrigeration to an extent also prevents putrefaction [63], neither of which were observed in our experiment.

Ca²⁺ levels were measured throughout the experiment to evaluate the potential preservative effects of EGEO and CFEO in brined pork contaminated with *S. typhimurium*. Calcium is a crucial electrolyte involved in various biochemical processes, including microbial metabolism, cell wall integrity, and enzymatic activity [66, 67]. Therefore, monitoring calcium levels provides insight into possible interactions between the EOs, bacterial activity, and meat matrix composition over time. Table 4 summarizes the Ca²⁺ levels results in the brines.

**Table 4:** Ca²⁺ concentration (mg/dL) in inoculated brined meat samples over time (mean ± SD)

Time (Days)	Saline Solution	EGEO	CFEO
1d	2.17 ± 0.67	1.63 ± 1.51	2.27 ± 0.36
3d	1.50 ± 0.20	1.87 ± 0.30	3.20 ± 0.35
7d	4.50 ± 4.56	2.23 ± 0.50	3.57 ± 0.29

Ca²⁺ concentration (mg/dL) in brine samples over time. Mean and standard deviation (SD) values were calculated from triplicate samples for each condition. Ca²⁺ levels were measured in the saline group (brine without EOs), EGEO group (brine with *Eucalyptus globulus* essential oil at 10× MIC), and CFEO group (brine with *Cymbopogon flexuosus* essential oil at 10× MIC) at days 1, 3, and 7 of storage at 8 °C ± 2 °C.

On day 1, Ca²⁺ concentrations were relatively stable across all experimental groups, with no significant deviations observed between the control (saline solution) and the treatment groups (brine containing EGEO or CFEO). This suggests that the initial addition of EOs did not immediately alter Ca²⁺ solubility or availability in the brining medium. By day 3, a slight decrease in Ca²⁺ levels was observed in the control group, likely due to interactions with proteins and other meat components, leading to precipitation or binding. Interestingly, both EO-treated groups exhibited a more stable Ca²⁺ profile, which could indicate a protective effect of EGEO and CFEO in maintaining electrolyte balance in the brining solution. One possible explanation is that the antimicrobial activity of the EOs reduced bacterial and endogenous enzymatic autolytic processes that may otherwise contribute to calcium depletion [62, 63, 67].

On day 7, a more pronounced reduction in calcium concentrations was noted in the control group, while the EO-treated samples maintained significantly higher levels. The decrease in the control may be associated with increased bacterial growth and metabolic activity, leading to calcium complexation or consumption. In contrast, the EO-treated groups demonstrated a preservation effect, possibly by limiting bacterial colonization and metabolic alterations in the meat environment. Notably, CFEO appeared to exert a stronger stabilizing effect on calcium levels compared to EGEO, suggesting potential differences in their mechanisms of action.

In meat, including cuts like sirloin, anoxic conditions promote quick degradation of oxygen dependent proteins (e.g. myoglobin, hemoglobin) and under no conservation methods, proteins that serve as ionic pumps also start to be hydrolyzed. ATPase, calsequestrins and other proteins involved in Ca²⁺ transport promote great ionic exchange in early *postmortem* changes, leading to an increase in Ca²⁺ ions [66, 68]. In fact, there is an increase in all biological cations, but especially Mg2+, Ca²⁺ and Na+ [69]. Cationic force increase is also related to water loss in meat tissues, which causes homeostatic ion imbalances [68, 69].

These findings support the hypothesis that EGEO and CFEO contribute to the stabilization of electrolyte balance during meat preservation, likely through their antimicrobial properties [70]. The ability of EOs to mitigate Ca²⁺ fluctuations could enhance the overall effectiveness of brining as a preservation method, reducing bacterial-induced deterioration and maintaining meat quality for extended storage periods.

Table 5 summarizes the Mg²⁺ levels across the three brines tested. Similarly to Ca²⁺, Mg²⁺ plays several crucial roles in meat cells, primarily related to cellular metabolism, enzyme activation, and structural stability. Mg²⁺ helps maintain energy production in muscle cells even *post-mortem*, influencing biochemical processes like glycolysis and pH regulation in meat [66–68].

**Table 5:** Mg²⁺ concentration (mg/dL) in inoculated brined meat samples over time (mean ± SD)

Time (Days)	Saline Solution	EGEO	CFEO
1d	1.60 ± 0.87	1.13 ± 0.78	1.07 ± 0.73
3d	3.67 ± 1.54	2.67 ± 0.86	0.93 ± 0.85
7d	1.80 ± 1.04	3.07 ± 0.61	3.33 ± 1.59

Mg²⁺ concentration (mg/dL) in brine samples over time. Mean and standard deviation (SD) values were calculated from triplicate samples for each condition. Mg²⁺ levels were measured in the saline control group (brine without EOs), EGEO group (brine with *Eucalyptus globulus* essential oil at 10× MIC), and CFEO group (brine with *Cymbopogon flexuosus* essential oil at 10× MIC) at days 1, 3, and 7 of storage at 8 °C ± 2 °C.

Mg²⁺ levels in the brine samples showed notable variations over time across the different experimental groups. On day 1, the Mg²⁺ concentration was relatively low in all groups, with the saline control (1.60 mg/dL) having a slightly higher concentration than the essential oil treatments (1.13 mg/dL for EGEO and 1.07 mg/dL for CFEO). This suggests an initial stabilization phase in which the electrolyte balance remains unaffected by the addition of EOs.

By day 3, a sharp increase in Mg²⁺ concentration was observed in the saline group (3.67 mg/dL), while the EGEO group showed a more moderate rise (2.67 mg/dL). Interestingly, the CFEO group exhibited the lowest Mg²⁺ levels (0.93 mg/dL), suggesting a possible interaction between CFEO and the mineral content in the brine. This reduction may be due to chelation, precipitation, or absorption of Mg²⁺ by components in CFEO.

At day 7, the Mg²⁺ concentration in the saline solution decreased to 1.80 mg/dL, potentially due to precipitation or protein-mineral interactions. In contrast, both EO-treated groups exhibited higher levels of magnesium compared to earlier time points, with EGEO reaching 3.07 mg/dL and CFEO reaching 3.33 mg/dL. This suggests that the EOs may have influenced Mg²⁺ retention or release within the brine system over time, potentially altering ionic equilibrium and impacting the preservation process.

**Fig. 8:**
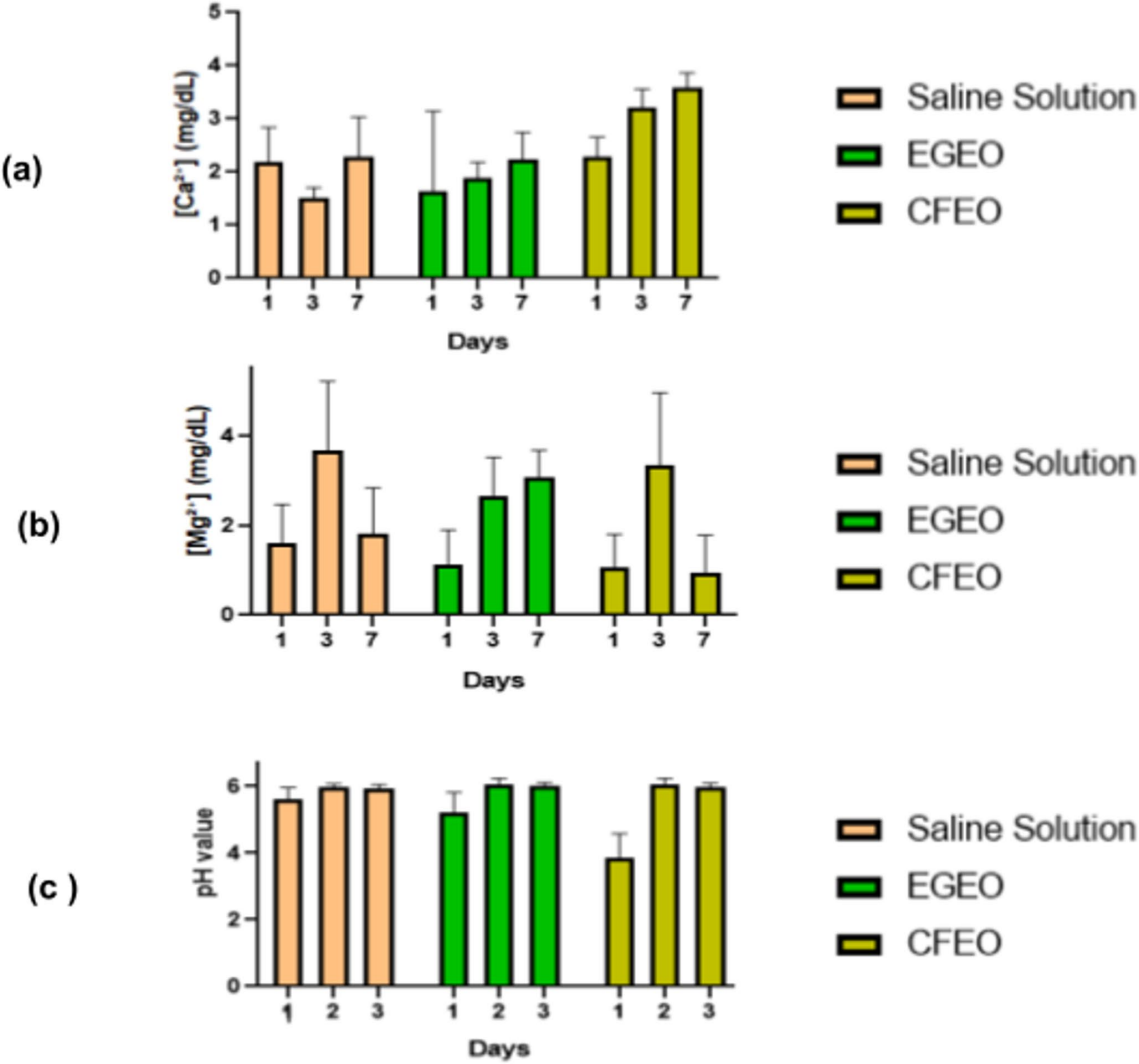
Changes in calcium (Ca²⁺) and magnesium (Mg²⁺) concentrations and pH values over time in meat samples treated with *Eucalyptus globulus* (EGEO) and *Cymbopogon flexuosus* (CFEO) essential oils compared to a saline solution control. **a** Ca²⁺ concentration (mg/dL) measured at different time points (days 1, 3, and 7). **b** Mg²⁺ concentration (mg/dL) across the same time points. **c** pH variation throughout the experiment. Error bars represent standard deviations (SD). Statistical significance was determined using Two-way ANOVA followed by Tukey’s post-hoc test. Were considered significant the differences between groups at each time point where *p* < 0.05

The contrasting trends between CFEO and EGEO treatments highlight the distinct chemical interactions these EOs may have with electrolytes. CFEO initially reduced Mg²⁺ concentration, while EGEO displayed a steadier increase. This difference could be linked to their respective chemical compositions, which may influence solubility, binding affinity, or metabolic activity in the brined meat matrix. Figure 8 shows the differences between pH and electrolytes levels in the samples.

The variations in pH across different treatments appear to be directly linked to Ca²⁺ and magnesium Mg²⁺ concentrations, given their roles in biological buffering systems and interactions with hydrogen ions [71, 72]. The initially lower pH in CFEO-treated samples compared to the brine and EGEO treatments suggests a greater availability of hydrogen ions, likely influenced by the chelating properties of some CFEO components, such as citral [73]. Over time, the pH stabilized across all treatments, reflecting a dynamic equilibrium between hydrogen ion concentration and electrolyte balance.

Ca²⁺ and Mg²⁺ play crucial roles in pH homeostasis by participating in biochemical equilibria [72, 74]. The increased Ca²⁺ levels observed in CFEO-treated samples may indicate leaching from the meat matrix or a compensatory response to acidification, as Ca²⁺ is often mobilized to counteract pH drops. In contrast, fluctuations in Mg²⁺ levels, particularly in the EGEO treatment, suggest a different interaction mechanism, possibly involving ionic exchanges that contributed to pH stability.

When correlating these biochemical findings with microbiological data, a possible explanation emerges. CFEO-treated samples exhibited a highly acidic environment on day 1 (mean pH ~ 3.86), which may have initially suppressed bacterial growth. However, as the pH increased (~ 5.99 on day 7), conditions became more favorable for *Salmonella* survival. Meanwhile, EGEO-treated samples maintained a more stable pH (~ 5.53 on day 1 to ~ 6.04 on day 7), potentially supporting a more sustained antibacterial effect.

Electrolyte measurements further revealed key trends. Ca²⁺ and Mg²⁺ levels were lower in CFEO-treated samples compared to EGEO and saline groups. Since these cations are essential for bacterial cell wall stability and stress responses, their depletion may have initially weakened bacterial resistance. However, as ionic disruption diminished over time, CFEO’s antimicrobial efficacy also declined.

## Conclusions

This study demonstrated that both EGEO and CFEO exhibit antimicrobial activity against *S.* typhimurium, although with distinct potency and biological effects. CFEO showed lower MIC and MBC values and rapid bactericidal activity in time-kill assays, indicating strong membrane-disruptive potential under controlled in vitro conditions. In contrast, EGEO displayed a comparatively higher MIC/MBC but induced notable alterations in bacterial metabolic behavior, including inhibition of H₂S production, suggesting possible interference with sulfur metabolism. These interpretations, however, should be considered mechanistic hypotheses supported by biochemical observations rather than definitive mechanistic conclusions.

Cytotoxicity assays provided valuable insights into the safety of these EO for food applications. Both oils exhibited varying degrees of cytotoxicity, with EGEO displaying lower cytotoxicity and preserved cell viability at higher concentrations. These findings suggest that while both EOs are effective in antimicrobial activity, their safety profiles differ, making CFEO a more potent antimicrobial but slightly less suitable for direct application due to its higher cytotoxicity.

In the in situ pork model, both essential oils were evaluated as adjuncts to a conventional brining system. As the saline control also resulted in complete inhibition of *Salmonella* spp., the antimicrobial effect cannot be attributed exclusively to the EOs. Rather, the findings suggest that EGEO maintained antimicrobial stability within the tested preservation matrix, whereas CFEO showed reduced long-term effectiveness. Therefore, the role of these essential oils should be interpreted as complementary to established preservation methods rather than as stand-alone preservative agents.

Overall, the results reinforce the antimicrobial potential of EGEO and CFEO and highlight their distinct biological interactions with bacterial cells and food matrices. Future studies should further investigate their mechanisms of action and evaluate optimized formulations and safety parameters to better define their applicability in food preservation systems.

## Supplementary Information

Below is the link to the electronic supplementary material.

Supplementary Material 1
