# Supplementary material for: Eucalyptus globulus and Cymbopogon flexuosus essential oils antimicrobial and conservative effects against Salmonella enterica serovar typhimurium and possible cytotoxicity: an in vitro and in situ investigation in inoculated pork sirloin
Source: Braz J Microbiol. 2026 Apr 27;57(1):124. doi: 10.1007/s42770-026-01923-x (PMC13121662; doi:10.1007/s42770-026-01923-x)

**Supplementary material**

**Supplementary figure 1.** Gas chromatography-tandem mass spectrometry (GC-MS) of (a) EGEO and (b) CFEO. The numbered peaks in each chromatogram correspond to the main compounds identified, whose chemical structures were obtained from PubChem and are displayed in the figure. For EGEO (a): Peak 1 – α-pinene; Peak 4 – eucalyptol; Peak 5 – γ-terpinene; Peak 7 – α-terpineol. For CFEO (b): Peak 3 – geranyl acetate; Peak 8 – neral; Peak 11 – α-citral; Peak 13 – D-limonene. Peak numbers in the chromatograms indicate the retention order of detected compounds.


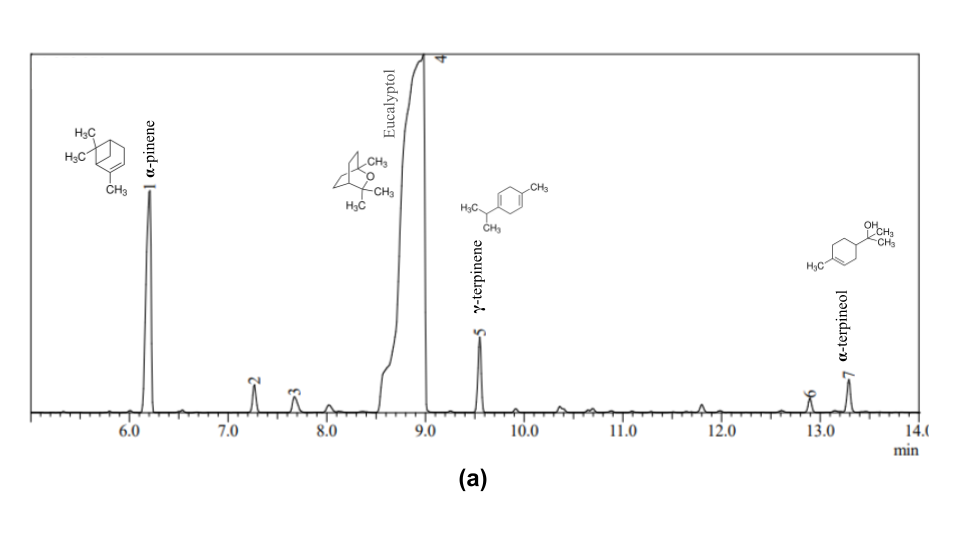


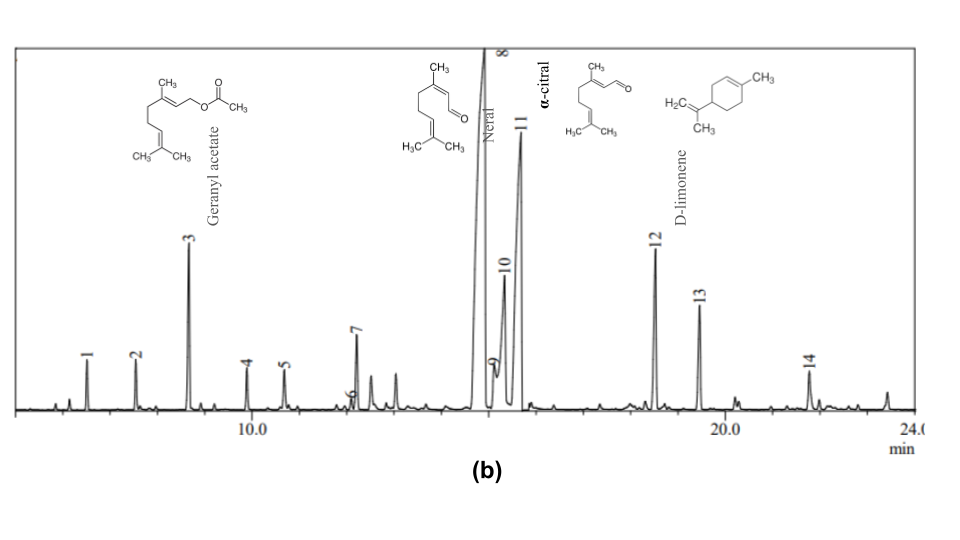

Supplement: Supplementary file 1 — Supplementary Material 1 [file 42770_2026_1923_MOESM1_ESM.docx]
